# *Taenia solium*, *Taenia saginata*, *Taenia asiatica*, their hybrids and other helminthic infections occurring in a neglected tropical diseases' highly endemic area in Lao PDR

**DOI:** 10.1371/journal.pntd.0006260

**Published:** 2018-02-08

**Authors:** Marcello Otake Sato, Megumi Sato, Tetsuya Yanagida, Jitra Waikagul, Tiengkham Pongvongsa, Yasuhito Sako, Surapol Sanguankiat, Tipparayat Yoonuan, Sengchanh Kounnavang, Satoru Kawai, Akira Ito, Munehiro Okamoto, Kazuhiko Moji

**Affiliations:** 1 Department of Tropical Medicine and Parasitology, Dokkyo Medical University, Mibu, Tochigi, Japan; 2 Graduate School of Health Sciences, Niigata University, Niigata, Niigata, Japan; 3 Joint Faculty of Veterinary Medicine, Yamaguchi University, Yamaguchi, Japan; 4 Department of Helminthology, Faculty of Tropical Medicine, Mahidol University, Bangkok, Thailand; 5 Station of Malariology, Parasitology, and Entomology of Savannakhet Province, Savannakhet, Lao PDR; 6 Department of Parasitology, Asahikawa Medical University, Asahikawa, Hokkaido, Japan; 7 National Institute of Public Health, Ministry of Health, Vientiane, Lao PDR; 8 Primate Research Institute, Kyoto University, Aichi, Japan; 9 Graduate School of International Health Development, Nagasaki University, Nagasaki, Japan; Instituto de Investigaciones Biomédicas, UNAM, MEXICO

## Abstract

Most part of Southeast Asia is considered endemic for human-infecting *Taenia* tapeworms; *Taenia solium*, *T*. *saginata*, and *T*. *asiatica*. However, until now there was no report of the occurrence of human cases of *T*. *asiatica* in Lao PDR. This study, conducted in Savannakhet Province, Lao PDR, microscopically examined a total of 470 fecal samples by Kato Katz method and found 86% of people harboring at least one helminth. Hookworms were detected in 56% of the samples besides *Opisthorchis* like eggs (42%), *Trichuris trichiura* (27%), *Ascaris* spp. (14%), and *Taenia* spp. (4%) eggs. Serology for cysticercosis showed 6.8% positives with results varying from 3% to 14.3% in Ethnic School students and Kalouk Kao village respectively. Species-specific PCR targeting mitochondrial DNA (mtDNA) of 28 tapeworms, recovered from 16 patients, revealed *T*. *solium* (n = 2), *T*. *saginata* (n = 21), and *T*. *asiatica* (n = 5). Two patients were confirmed to be coinfected with *T*. *saginata* and *T*. *asiatica*, indicating the endemicity of the 3 human *Taenia* in Lao PDR. However, nucleotide sequencing of a nuclear DNA gene, DNA polymerase delta (*pold*) revealed that all the tapeworms identified as *T*. *asiatica* using mtDNA had *T*. *saginata* type allele at *pold* locus, demonstrating that they are not “pure *T*. *asiatica*” but the hybrid descendants between the two species, confirming the wide distribution of hybrids of *T*. *saginata*/ *T*. *asiatica* in Southeast Asia. The high prevalence of several helminthic NTDs in east Savannakhet area even with conventional control measures indicates the importance to establish wide and multifaceted health programs to sustainably improve the quality of life of the populations living in these communities.

## Author summary

Southeast Asian Countries are endemic for several foodborne and soil-transmitted helminths occurring in different levels and areas, depending on environmental and cultural conditions. This study aimed to study the soil-transmitted helminths (STHs) and foodborne parasites in Savannakhet Province of Lao PDR, bordering with Vietnam. We found people infected with hookworms, roundworms, whipworms, intestinal/liver flukes, and tapeworms. We also detected antibodies against cysticercosis, an infection caused by eating the eggs of *Taenia solium*, the pork tapeworm. Focusing on human tapeworm infection, using molecular techniques based on mitochondrial DNA, we detected the three species of human tapeworms *T*. *solium*, *T*. *saginata* and *T*. *asiatica*. Interestingly, when we checked the same material using nuclear gene markers we noted that *T*. *asiatica* found in the region were the hybrid descendants of *T*. *saginata* and *T*. *asiatica*. The causes of hybridization may include people harboring different tapeworm’s species at the same time, allowing the exchange of genetic material but, the consequences of this hybridization are unknown including the seriousness of disease in people. Despite the deworming programs, there is a high prevalence of STHs and foodborne parasites in east Savannakhet area, therefore wide and sustainable health programs are an urgent task to improve the quality of life of the people living in the area.

## Introduction

Only human are the definitive hosts for *Taenia solium*, *Taenia saginata*, and *Taenia asiatica*, which are referred as the human-*Taenia*. The distribution of each of the 3 species of human *Taenia* depends on peoples’ cultural characteristics which involve the consumption of undercooked meat or organs of intermediate hosts infected with viable metacestodes [1–3]. Swine are the intermediate hosts for *T*. *solium* and *T*. *asiatica*. However, the metacestodes of these species present different tropism: usually muscle and brain for *T*. *solium*, and viscera, mainly liver, for *T*. *asiatica* [4–6]. Domestic bovine are the main intermediate hosts for *T*. *saginata* with cysticerci establishing predominantly in the muscles [7].

Southeast Asia is considered an endemic area for the 3 species of human *Taenia* with several reports of occurrence in human and animals [8–11]. However, there is no report of the occurrence nor evidence of *T*. *asiatica* in human in Lao PDR despite its localization, surrounded by endemic countries [12–14]. Antibody serosurveillance of four provinces in the northern area of Lao PDR in 2011 indicated high frequency of contacts with adult (46.7%) and larval parasites (66.7%) [14]. The existence of *T*. *solium* was confirmed by DNA sequencing of copro-PCR positive fecal samples, but no *T*. *saginata* or *T*. *asiatica* were detected [14]. Furthermore, in a recent study, 15 haplotypes of *T*. *saginata* were obtained from 30 isolates from Khammouane Province, central Lao PDR [15]. An extensive study on the prevalence of taeniasis in Lao PDR with whole country coverage reported the presence of mainly *T*. *saginata* found in all Lao PDR’s provinces and *T*. *solium* in Luang Prabang, northern area [10].

In this study, we report a high prevalence area for foodborne parasites and STHs. Furthermore, we could detect worm carriers of *T*. *solium*, *T*. *saginata* and *T*. *asiatica* by mitochondrial DNA in east Savannakhet Province, suggesting that Lao PDR as an endemic country for the 3 human-*Taenia* species. Moreover, we verified hybridization of *T. saginata* and *T. asiatica* is likely to be occurring in the region.

## Methods

### Study area and human sampling procedures

**Fig 1:**
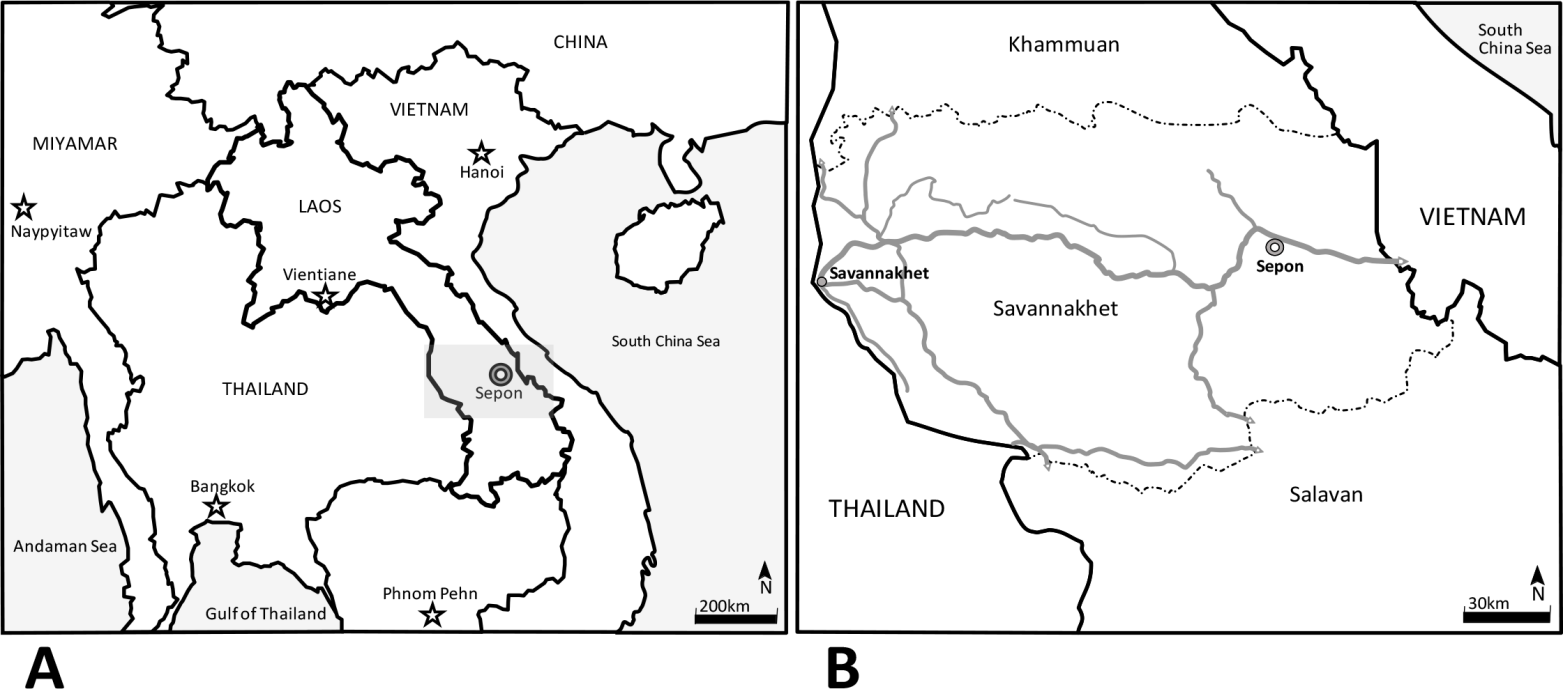
Map showing Laos localization and its neighboring countries in “A” a detailed map of Savannakhet province in “B” (gray square in “A”). Stars represents the localization of the capital cities of the countries in the map on the left (A). Double circle marks signalizes the localization of Sepon, the study area in A and B and shows the proximity of the city with Vietnam. In B, continuous gray lines represent the main roads of Savannakhet Province, dash-dot lines are the borders of the provinces of Khammuan (north) and Salavan (south) and, continuous black lines are the Laos-Thailand and Laos-Vietnam borders in west and east respectively.

The study was conducted in Sepon District, Lao PDR in March and December 2013. The area is located in the eastern part of Savannakhet province and is bordering with Quang Tri province of Central Vietnam (Fig 1) and it is covered by subtropical forests in its majority. Ancient human occupation is reported in the actual area of Lao PDR, and according to the last classification, there are 49 different ethnical groups in the country, with more than 4 groups living in Savannakhet area [16]. The participants joined the study on a voluntary basis, from an estimated population reported by the Basic Health Center of Sepon district of 743 people in the study area, 396 (53%) males and 347 (47%) females aged from 3 to 74 years old, and living in the 3 studied villages (Kalouk Kao, Poung, and Ayay Yay) besides the residents of an ethnic college located in Sepon district area, at the eastern Savannakhet province (Fig 1). A detailed explanation of the study was done in the local language for proper understanding. Adult subjects provided written informed consent, and a parent or guardian of any participant child provided informed consent on the child’s behalf and, after approving the informed consent, the participants received instructions for collecting and transporting the fecal samples. Fecal examinations were conducted at the Sepon District Hospital by Kato-Katz modified cellophane thick smear method (KK) [17]. Each slide was examined under a microscope, helminth eggs were counted, and the number of eggs per gram of feces (EPG) was calculated as previously described [18]. Blood sampling was conducted from 235 persons for serological diagnosis of cysticercosis. Feces and serum samples were then brought back to the laboratory in Thailand and Japan for further analysis.

### Copro PCR

Fecal samples were submitted to KK and molecular procedures. Samples for copro PCR were added sufficient volume of RNAlater stabilization solution (Life Technologies, USA) and brought to the laboratory for Copro PCR analysis. Copro DNA technique was done as previously established [19] with some modification, briefly; fecal samples were homogenized, the volume estimated and 300mg of each sample was used for DNA extraction. The fecal material was disrupted with a μT-12 beads crusher (TAITEC Co., Koshigaya, Japan) using 3 stainless steel beads of 4mm plus 200mg of 0.2mm glass beads in each tube. DNA was extracted from the homogenized solution using the QIAamp DNA Stool Mini Kit (QIAGEN, Hilden, Germany) following the manufacturer’s instructions. Final DNA elution was done in 30 μl of elution buffer.

PCR was conducted using a T100 Thermal Cycler DNA thermocycler (BIO-RAD, Hercules, CA, USA). The reaction was carried out in a final volume of 25 μl containing PCR reagent (TOYOBO, Osaka, Japan) and 1 `l of DNA preparation as template. The DNA samples were initially denatured at 94°C for 4 min, followed by 30 amplification cycles of denaturation at 94°C for 1 min, annealing at 60°C for 30 s, and elongation at 72°C for 2 min. PCR products were electrophoresed on a 2.0% agarose gel with positive samples showing amplicons of the proper size for each *Taenia* species [19].

### Anthelmintic treatment and collection of expelled worms

The participants presenting *Taenia* eggs on KK or voluntarily looked-for treatment were given a single oral dose of niclosamide (Yomesan, Bayer AG, Germany) according to the fabricant recommendations, followed by purgation with 60 ml of saturated magnesium sulfate solution. After treatment, attention for worm expulsion was dispensed for all participants, including the people who showed the *Taenia* eggs in fecal examination or the people who requested treatment even with no positive results in KK exams [20]. Discharged parasites recognizable by naked eye were separated, identified, washed in saline solution (NaCl 0.85% w/v) and preserved individually in 70% ethanol or RNAlater stabilization solution (Life Technologies, USA). The samples were transferred to a laboratory in Japan for further DNA analysis.

### DNA extraction, PCR analysis, and sequencing of adult worms

The genomic DNA of each ethanol preserved tapeworm was extracted using QIAamp DNA Mini Kit (Qiagen) and subsequently used as a template for polymerase chain reaction (PCR). For the differentiation of three human *Taenia* species, multiplex PCR was performed as previously described [19], with a slight modification. Briefly, one reverse and four forward primers were used to amplify different sizes of amplicons, specific for the cytochrome *c* oxidase subunit I (cox1) gene sequences of *T*. *solium* Asian genotype, *T*. *solium* Afro-American genotype, *T*. *saginata* and *T*. *asiatica*, respectively. The forward primer specific to *T*. *asiatica* was newly designed as TasiMpF (5’- TTA TTT ATT TAC GTC AAT CTT ATT G -3’), instead of the originally used primer.

For some of the tapeworms identified as *T*. *saginata* or *T*. *asiatica*, nucleotide sequencing of nuclear DNA gene markers was performed to examine whether they are the hybrid descendants of the two *Taenia* species. Partial sequences of two nuclear genes, ezrin-radixin-moesin-like protein (*elp*) and DNA polymerase delta (*pold*), were amplified by PCR and directly sequenced [21,22]. In the case of double peaks in the sequencing electropherogram, PCR products were cloned using pGEM-T vector (Promega) transformed into *Escherichia coli* DH5α and plated on LB agar containing X-Gal (20mg/ml) and ampicillin (100ug/ml). At least 10 positive clones from each PCR product were used for nucleotide sequence confirmation.

### Serodiagnosis for cysticercosis

ELISA and immunoblotting using LMWAgs were performed as previously described [23]. Briefly, for ELISA, 100 μl of 1 μg/ml of *T*. *solium* LMWAgs in PBS were loaded in 96-well microplates (Maxisorp, Nunc, Copenhagen) overnight at 4°C, blocked with 300 μl of blocking buffer (20 mM Tris–HCl, pH 7.6, 150 mM NaCl, 1.0% casein, 0.1% Tween 20) at 37°C for 1 hour and washed twice with PBS containing 0.1% Tween 20 (PBST). Serum samples diluted 1:100 in blocking buffer, were applied (100 μl/well) in duplicates and incubated at 37°C for 1 h. After washing twice in PBST, the plates were incubated with 100 μl/well of recombinant protein G conjugated with peroxidase (Invitrogen, USA) diluted 1:2000 in blocking buffer at 37°C for 1 h. For color development, the plates were incubated with 100 μl of peroxidase substrate ABTS (2,2’-azino-di(3-ethyl-benzothiazoline-6-sulfonate)) (Sigma-Aldrich) in 0.2 M citric acid buffer (pH 4.7) for 30 min at room temperature. Optical density (OD) was determined at 405 nm for each well using microplate reader (Immuno Mini NJ-2300, Biotec, Japan). The cut-off value was determined as the mean of OD plus 4 standard deviations of sera from 37 healthy donors.

Immunoblot was used for confirmation of the ELISA positive serum samples and serum samples with OD value close to the cut-off value. Briefly, LMWAgs (60 μg/mini gel) in SDS sample buffer (62.5 mM Tris-HCl, pH 6.8, 2.0% SDS, 50 mM dithiothreitol and 10.0% glycerol) were loaded in a 15.0% polyacrylamide gel. The separated proteins were transferred onto a polyvinylidene difluoride (PVDF) membrane sheet (Millipore) and then blocked with blocking buffer. Serum samples diluted 1:20 in blocking buffer were incubated at room temperature for 1 h. After washing 3 times for 5 minutes in blocking buffer, the membranes were incubated with the peroxidase-conjugated recombinant protein G (Invitrogen, USA) diluted 1:2000 in blocking buffer. The substrate (4-Chloro-1-Naphthol/Phosphate) was used for color development at room temperature for 30 min, and the reaction was stopped by washing with water.

### Data analysis

All the data collected in paper forms were tabulated and the analyzed in Excel 2016 (Microsoft) and EpiInfo version 7.1.5.0 (Centers for Diseases Control and Prevention, Atlanta, GA, USA). DNA sequencing data were edited and analyzed using MEGA 6 software [24]. In this cross-sectional study, the sample size was determined at a confidence level of 95% with confidence limits of 5% considering the entire population, gender and age calculated with the expected frequencies of cysticercosis and taeniasis. The data were analyzed using descriptive statistics and chi-square test to determine association between the results of the tests used in each category of data. P values <0.05 were considered statistically significant.

### Ethics

This study was approved by the National Ethical Committee for Lao Health Research of the Ministry of Health, Lao PDR (172/NECHR).

## Results

The participants of this study that provided fecal samples totalized 470 individuals (259 males and 211 females aging from 5 to 72 years old), corresponding to 63% (470/743) of the total estimated population, with fecal samples and 235 individuals out of 743 (32%) provided blood samples.

The information provided from Sepon Health Center regarding the cultural aspects of the region showed no restrictions for food within the ethnic groups living in the area, with all the analyzed population consuming different types of meat and vegetables including raw or cooked beef and pork meat or viscera and cooked and fresh vegetables mainly produced for subsistence in the local area or acquired from local markets. The residents of the ethnical college were from different ethnical groups (Makong, Tri, Katang, Ta Oi). The students enrolled in this study totalized 66 people with serum and fecal samples, 32% (16) female and 68% (50) male, were aged from 10 to 19 years old. The students preserved their cultural traditions while using the infrastructure provided by the school and frequently went back to their hometown, located in different areas of Sepon district.

### Fecal examination (KK), copro-PCR and tapeworm expulsion

Stool samples analysis by KK were performed on 470 fecal samples. Analyses of the results revealed 86% of people harboring at least one species of parasite: 42% (196/470), 31% (146/470), 11% (50/470) and 2% (11/470) harbored 1, 2, 3 and 4 helminth species, respectively. The prevalences of the detected helminth eggs were 56% (265/470) for hookworms, 42% (199/470) for *Opisthorchis* like eggs, 27% (129/470) for *Trichuris trichiura*, 14% (66/470) for *Ascaris* spp., and 4% (19/470) for *Taenia* spp.

Copro-PCR to detect human *Taenia* DNA were performed in 163 fecal samples revealing 9.8% (16/163) of *T*. *saginata*, 3.1% (5/163) of *T*. *solium* and 1.8% (3/163) *T*. *asiatica*, indicating the endemicity of the 3 human *Taenia* in the Sepon district.

Eighteen people received treatment with niclosamide, a total of 28 tapeworms were recovered from 16 taeniasis patients in 4 villages and the ethnic school. Two worms were identified as *T*. *solium* and 26 worms appeared to be *T*. *saginata* or *T*. *asiatica* morphologically. All the specimens were submitted to DNA examination. People receiving treatment for taeniasis and seropositives for cysticercosis by village, tapeworm expulsion and the results of the different diagnostic tests done are shown in Table 1.

**Table 1:** The tapeworm expulsion and the results of the different diagnostic tests done in Sepon district, Savannakhet province, Lao-PDR. People receiving treatment for taeniasis and seropositives for cysticercosis is listed by village. Eighteen people received treatment for tapeworms with a total of 28 worms recovered from 16 patients. Kalouk Kao and Poung presented significantly more taeniasis than Ayay Yay and the Ethnic School students. *Taenia asiatica* diagnosed by mitochondrial DNA was recovered only from Kalouk Kao where 2 people harbored concomitant infection by *T*. *saginata* and *T*. *asiatica*.

Village	Gender	Age	KK					ELISA	Copro PCR	Deworming (number)
			OvL	Tae	Hw	Al	Tt			
Kalouk Kao	F	7	-	-	+	-	+	+	Nd	
	F	12	-	-	-	-	+	-	*T*. *solium*	
	M	18	-	+	-	-	-	Nd	*T*. *solium*	*T. solium* (1)
	M	32	+	+	+	-	-	-	-	*T*. *saginata* (2), *T*. *asiatica* (1)
	M	32	-	-	-	+	-	-	*T*. *saginata*	
	F	37	+	-	-	+	+	+	Nd	
	M	39	+	-	-	-	+	+	-	
	M	41	+	+	+	-	+	-	*T*. *asiatica*	*T*. *asiatica* (3)
	M	44	-	+	-	-	+	-	Nd	*T*. *saginata* (1), *T*. *asiatica* (1)
	M	49	+	-	-	-	-	+	-	
	F	54	-	-	+	-	+	+	Nd	
	M	67	-	+	-	-	-	+	-	*T*. *saginata* (1)
	M	68	-	-	-	-	-	+	Nd	
Poung	F	6	-	-	-	-	-	Nd	*T*. *saginata*	
	F	8	-	-	+	-	-	Nd	*T*. *saginata*	
	F	9	-	-	+	-	-	Nd	*T*. *saginata*	
	M	9	-	-	+	-	-	Nd	*T*. *solium*	
	F	9	-	-	+	-	-	+	Nd	
	F	20	+	-	+	-	-		*T*. *saginata*	
	M	22	-	-	+	-	-	Nd	*T*. *saginata*	
	M	25	-	-	-	-	-	+	Nd	
	M	26	-	-	+	-	-	Nd	*T*. *saginata*	
	M	27			Nd			+	Nd	*T*. *saginata* (4) #
	M	27	+	-	-	-	-	+	Nd	
	M	28	-	+	+	-	-	-	*T*. *saginata*	no worm
	M	30			Nd			Nd	Nd	*T saginata* (1) #
	F	35	-	-	-	-	-	+	Nd	
	M	38	+	+	+	-	-	-	Nd	*T*. *saginata* (2)
	F	41	-	+	-	-	-	Nd	*T*. *saginata*	*T*. *saginata* (2)
	F	46			Nd			-	Nd	*T*. *saginata* (1) #
	M	46	+	-	-	-	-	-	*T*. *saginata*	
	M	47	-	+	+	-	-	-	Nd	*T*. *saginata* (2)
	M	48	+	-	+	-	-	+	-	
	F	49	+	-	-	-	-	Nd	*T*. *saginata*	
	F	55	-	-	+	-	-	-	*T*. *saginata*	
Ayay Yay	M	14	-	-	-	-	-	-	*T*. *saginata*	
	M	15			Nd			Nd	Nd	*T*. *saginata* (1) #
	F	15	-	+	-	-	-	Nd	-	*T*. *saginata* (1)
	M	16	-	-	+	-	+	-	*T*. *solium*	
	M	16	-	-	-	+	-	Nd	*T*. *saginata*	
	F	22	+	-	+	-	-	+	-	
	F	29	-	-	-	-	-	-	*T*. *asiatica*	
	M	31	+	-	+	-	-	Nd	*T*. *saginata*	
	M	37	-	+	-	-	-	Nd	Nd	*T*. *saginata* (2)
	F	40	+	-	+	-	-	+	-	
	M	40	+		+		+	-	*T*. *asiatica*	
	F	44	+	-	-	-	-	-	*T*. *saginata*	
	M	46	-	-	-	-	-	-	*T*. *solium*	
Ethnic School	M	12	+	-	+	-	-	+	Nd	
	M	12	+	+	+	-	-	-	Nd	no worm
	M	12	-	+	-	-	-	-	Nd	*T*. *solium* (1)
	M	15	-	-	-	+	-	+	Nd	
	M	19	-	+	-	-	-	Nd	Nd	*T*. *saginata* (1)

Eggs observed in KK: Ov-L = *Opisthorchis viverrini* Like; Tae = *Taenia* spp.; Hw = Hookworm; Al = *Ascaris lumbricoides*; Tt = *Trichuris trichiura*

Nd = not done

# = presented voluntarily for deworming

### DNA analyses from expelled tapeworms

Firstly, using multiplex PCR targeting mtDNA *cox1* gene, based on mtDNA sequences, 75% (21/28) of the tapeworms were identified as *T*. *saginata*, 18% (5/28) were *T*. *asiatica* and 7% (2/28) *T*. *solium*. Two patients from Kalouk Kao village harbored multiple tapeworms. One patient had a mixed infection with three tapeworms (two *T*. *saginata* and one *T*. *asiatica*). The other had two worms (one *T*. *saginata* and one *T*. *asiatica*). *T*. *asiatica* was confirmed only in Kalouk Kao village, and *T*. *solium* was found in Kalouk Kao village (n = 1) and in the Ethnic School (n = 1). Nuclear DNA analysis was carried out to clarify whether the tapeworms identified as *T*. *asiatica* by mtDNA were pure *T*. *asiatica* or hybrid descendants between *T*. *saginata* and *T*. *asiatica*. The tapeworms identified as *T*. *saginata* or *T*. *asiatica* in Kalouk Kao (n = 9) were used for nuclear DNA study.

Partial sequences of *elp* and *pold* genes (1164 bp and 1097 bp) were obtained from all except three samples by direct sequencing. After cloning and sequencing, two different nucleotide sequences were obtained from each of those three samples. In the *pold* locus, all the alleles obtained from nine tapeworms were *T*. *saginata* types (*pold*A or *pold*B) as previously found [22], and one worm was heterozygous of *pold* A/B alleles (Table 2). On the other hand, both *T*. *asiatica* type (*elp*A) and *T*. *saginata* type (*elp*C) alleles [21, 25] were confirmed in *elp* locus. The genotype of *elp* locus mostly corresponded to the species identification by mtDNA, however, two worms with *T*. *saginata* type mtDNA were heterozygous of *elp* A/C alleles (Table 2).

**Table 2:** Samples of the *Taenia* tapeworms used for molecular analysis. All the samples were obtained in Kalouk Kao. The allele shown in bold is *Taenia asiatica* type allele.

Sample ID	mtDNA type	Genotype at *elp* locus	Genotype at *pold* locus
13KK2771a	*T*. *saginata*	***elpA****/elpC*	*poldA/poldA*
13KK2771b	*T*. *saginata*	***elpA****/elpC*	*poldA/poldA*
13KK2771c	*T*. *asiatica*	***elpA****/****elpA***	*poldB/poldB*
13KK2804a	*T*. *saginata*	*elpC/elpC*	*poldA/poldB*
13KK2804b	*T*. *asiatica*	***elpA****/****elpA***	*poldB/poldB*
13KK2838	*T*. *saginata*	*elpC/elpC*	*poldB/poldB*
13KK2892a	*T*. *asiatica*	***elpA****/****elpA***	*poldB/poldB*
13KK2892b	*T*. *asiatica*	***elpA****/****elpA***	*poldB/poldB*
13KK2892c	*T*. *asiatica*	***elpA****/****elpA***	*poldB/poldB*

### Serological detection of cysticercosis

Serum samples collected totalized 235 samples, corresponding to 50% of the fecal samples, once not all the individuals that provided fecal samples accepted to provide blood samples.

**Fig 2:**
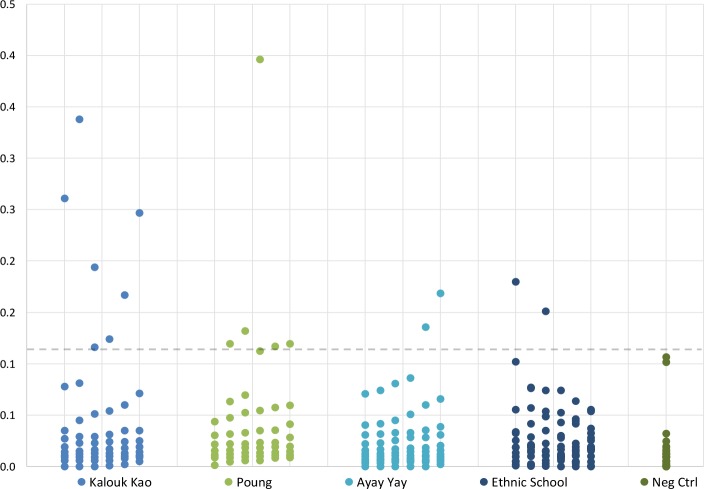
Results of ELISA for cysticercosis using LMWAgs in Sepon, Savannakhet, Laos. A total of 235 serum samples from 3 villages and the Ethnic School were tested. In total 7.2% (17/235) samples were considered cysticercosis positives in ELISA, confirmed by immunoblot. Kalouk Kao village presented the higher number of positives 14.3% (7/49), followed by Poung village with 10.7% (6/56). Ayay Yay village and the students of the Ethnic School in Sepon presented significant lower seropositivity to cysticercosis (p<0.05) with 2 positives each, corresponding to 3.1% (2/64) and 3.0% (2/66) of positivity respectively. Two *T*. *solium* tapeworms were confirmed from Kalouk Kao village (n = 1) and Ethnic School (n = 1). The cutoff value of 0.115 (dash line) was calculated as 4 times the mean OD of negative control samples (Neg Ctrl).

Results of ELISA confirmed by immunoblot showed the presence of positive cases of cysticercosis in all the villages with a prevalence of 7.2% (17/235) as shown in Fig 2. Considering the results by village, 2 locations, Kalouk Kao and Poung presented higher prevalence with 14.3% (7/49) and 10.7% (6/56) respectively. The serum samples from Ayay Yay village and the Ethnic School students presented lower seropositives to cysticercosis when compared to the other villages (p<0.05) with 2 cysticercosis positives samples each, corresponding to 3.1% (2/64) and 3.0% (2/66) of the studied people respectively.

## Discussion

### Ethnicity, general habits and parasitic infection in Sepon

This is the first report of *T*. *asiatica* in Lao PDR, a country with considerable differences in latitude from the south at 13^o^N in Champassack to Phongsali (Lat.22^o^N) the northern province, presenting great climatic diversity. The geographic particularities of the country, as the Boulavean plateau and the Mekong basin, created a basis for the development of different ethnicities, with many cultures and eating habits. A total of 49 ethnic groups have been recognized in Lao PDR [16], consequently, as one could expect, parasitic infection prevalence patterns also might differ according to different areas of the country and may explain some parasitic infection particularities as we observed in Sepon, now considered an endemic area for the three human *Taenia*. The absence of restrictions on food consumption can contribute to the parasite infection in those communities. Additionally, we detected rates as high as 86% of the studied population harboring at least one species of helminth. In general, there is a lack of sanitary infrastructure, toilet access and other issues like no schooling and basic hygiene, factors that would be considered causes for the high infection rates of parasitic infection, as pointed out in Lao rural communities [11].

### Cysticercosis education and hygiene

The villages of Poung and Kalouk Kao, where higher prevalence for all helminths was recorded, presented no structures for sewage treatment and the lowest education level according to the data provided by the Sepon Health Center. The lower prevalence of cysticercosis was found within the students, and it can be due to the access to education and basic infrastructure, as toilet, positive points on the protection for infection as previously reported [11], indicating that basic and general hygiene practices could diminish the infection levels in endemic areas. Another issue that could interfere with the knowledge or understanding of the importance of hygiene habits is the adhesion of the target population to health promotion initiatives, perceived when only 49 of 129 people listed collaborated with this study in Kalouk Kao village. That is, the village with less sanitary conditions presented the lowest adhesion to the health project, as we verified with only 38% of people joining the study. Bringing the interest of more people for health actions is an important issue to be improved in health programs for better results.

The occurrence of cysticercosis in the studied area was detected by serology without additional supportive data. Despite no clinical symptoms of neurocysticercosis were reported by the health center staffs or the villagers, suggesting there might be non-clinical cysticercosis cases, follow-up studies for the confirmation of such cases is an urgent task for early evaluation and treatment.

### Intrinsic relationship between *T*. *saginata* and *T*. *asiatica*

In the present study, all the three human *Taenia* were confirmed from taeniasis patients by the conventional PCR method targeting mtDNA. However, all the tapeworms identified as *T*. *asiatica* had *T*. *saginata* type *pold* allele, indicating that they are not “pure *T*. *asiatica*”. It has been shown that most of the tapeworms identified as *T*. *asiatica* using mtDNA have genetic traces of *T*. *saginata* in some nuclear DNA loci, and possible “pure *T*. *asiatica*” has been confirmed only in Taiwan and Philippines until now [21, 22, 25]. Those tapeworms showing nuclear-mitochondrial discordance are considered to be derived from the hybrid descendants between “pure *T*. *asiatica*” and “pure *T*. *saginata*”. Although the infection of *T*. *asiatica* in humans has been associated with eating raw or undercooked pork viscera, it is uncertain which animals, cattle and/or pigs, are the intermediate hosts of the hybrids. To clarify the host affinity and tissue tropism of *T*. *saginata*, *T*. *asiatica*, and their hybrids, it is necessary to examine the cysticercus from domestic animals by using both mtDNA and nuclear gene markers.

The reasons for the endemicity of the hybrids between *T*. *asiatica* and *T*. *saginata* in this region could be the proximity and the commuting of people and goods from Vietnam, a reported endemic area for *T*. *asiatica* with several human cases [26, 27]. Ethnic overlapping occurs on all borders of Lao PDR with neighboring countries especially the Austro-Asiatic groups on both sides of the Laos-Vietnam border as well as the cultural behavior of ethnical communities which are overlapping in this Laos-Vietnam border region [16] with habits of eating pork liver in several dishes. Moreover, in the west area of Savannakhet province which is bordering with Thailand, also a known endemic country for *T*. *asiatica* [9], there are reports for *T*. *saginata* and *T*. *solium* only [10, 28] reinforcing the idea of the Laotian *T*. *asiatica* or its hybrids origin from Vietnam, though more studies in this issue are necessary.

The causes of hybridization may include people harboring different tapeworm’s species as observed in this (Table 1) and other studies [21, 22, 25]. Multi-species parasitism may facilitate the exchange of genetic material allowing the occurrence of hybrids. However, the consequences of this hybridization as the seriousness of disease in people and even if the hybrid descendants can produce viable generations are unknown. For further studies, to detect hybrid cysticerci in intermediate hosts, confirm hybridization, and its infectivity to intermediate hosts is necessary.

### MDA, diagnosis and the persistence of taeniasis and other neglected tropical diseases

MDA is the recommended strategy of the World Health Organisation to control or eliminate NTDs in endemic areas. It has been implemented widely in Southeast Asian countries and its success is related to the improvement of sanitation and education programs [29]. The Sepon region is included in the MDA programs for elimination of parasitic diseases with treatment using praziquantel. The detection of seropositive individuals for cysticercosis leads to a point of concern in this issue: the occurrence of seizures and other collateral effects after treatment due to the inflammation caused by the sudden damage or death of cysts in the brain [30–33]. Side symptoms after treatment, including seizures, were reported by the population submitted to praziquantel MDA in Lao PDR, leading the people to reject subsequent treatments and the stop of the program. Nowadays only children under 5 years of age will take mebendazole when going for vaccination at primary school under a WHO project (Dr. Pongvongsa personal communication). The strict calculation of doses to be administrated should be considered in prevalent areas for cysticercosis, furthermore, a program for early diagnosis of cysticercosis in asymptomatic patients could be designed in these areas for improvement of MDA as accidental death after treatment may occur due to the extensive use of praziquantel in MDA of Asian countries [34].

Another issue in this endemic area for 3 species of human *Taenia* is to detect worm carriers. As we could observe in the results of worm recovering after treatment, the number of worm carriers would be higher if we combine the results of KK, Copro-PCR, and self-detection (Table 1). Unfortunately, Copro-PCR was not done in the field, so its results were not used for treatment protocols. Therefore, copro-PCR is a feasible option to be done in province’s central laboratories/health centers, where a safe treatment can be prescribed and conducted. In this study, we could note self-diagnosis as an educational alternative for detection of worm carriers; after the explanation of the study and the description of the fecal aspects and symptoms, 4 individuals who could not supply fecal samples, voluntarily came to expel worms, with 100% of worm expulsion (Table 1). This could be an excellent method for detection of worm carriers with a high rate of success as reported in a Mexican endemic area for *T*. *solium* [35].

Differently from the other studies in the central, north and northeastern areas of Lao PDR, where cases of *T*. *asiatica* were not found [12, 14, 15], in the east part of Savannakhet province (Fig 1) we could detect the presence of 3 species of human *Taenia* species in Sepon district that border Vietnam. The prevalence of *Taenia* eggs in KK was not high as presented by Okello et al. [14] which detected percentages as high as 46%. This result raises suspicion that other "hotspots" of *T*. *solium* hyper endemicity may exist in some regions, particularly in communities where the consumption of raw or undercooked pork is common, associated with the lack of health education.

Regarding other helminths observed in this study, the high prevalence of *Opisthorchis* like eggs (43%) is also a point of concern once *Opisthorchis viverrini* infection is a major cause of cholangiocarcinoma in endemic areas [36]. *O*. *viverrini* is a fishborne fluke and, like *Taenia* and its infection to humans is related to the consumption of raw fish meat (cyprinid fish) [37] and to domestic dogs, natural reservoirs of *O*. *viverrini*, which can be an important source of aquatic environment contamination because of its routine behavior to assess water sources [38]. Moreover, a high prevalence of hookworm (57%) was found, a parasitic disease where dogs play an important role [38, 39]. Dog meat is eaten in Asian countries including Lao PDR. However, considering the dog as an intermediate host of *T*. *solium* [40], dogs survey for cysticercosis in addition to pigs’ survey may also be important to screen the risk factor for human infections. More studies on the ecological aspects of NTDs, as carried out in other localities including checking reservoir animals and using environmental DNA detection [38, 41], would be an interesting way to determine the level of exposure of the people living in endemic areas to agents causing diseases like *O*. *viverrini*, hookworms and other STHs found in this study as *Ascaris* and *Trichuris*.

### Conclusion

This study revealed a highly endemic area for helminthic diseases in east Savannakhet, Lao PDR including the high occurrence of STHs and foodborne trematodes. The existence of *T*. *solium* in Savannakhet province was confirmed in this study, moreover hybrids descendants between *T*. *saginata* and *T*. *asiatica* were detected, indicating the presence of 3 human-*Taenia* species in Lao PDR. The situation points out the importance of establishing new, wide and multifaceted health program to sustainably improve the quality of life of the populations living in these communities.

## Supporting information

S1 Checklist STROBE checklist. (DOC)
