# Supplementary material for: Taenia solium, Taenia saginata, Taenia asiatica, their hybrids and other helminthic infections occurring in a neglected tropical diseases' highly endemic area in Lao PDR
Source: PLoS Negl Trop Dis. 2018 Feb 8;12(2):e0006260. doi: 10.1371/journal.pntd.0006260 (PMC5821399; doi:10.1371/journal.pntd.0006260)
Supplement: S1 Checklist — (DOC) [file pntd.0006260.s001.doc]

STROBE Statement—Checklist of items that should be included in reports of ***cross-sectional studies***

|  | Item No | Recommendation |
| --- | --- | --- |
| **Title and abstract** | 1 | (*✓*) Indicate the study’s design with a commonly used term in the title or the abstract |
|  |  | (*✓*) Provide in the abstract an informative and balanced summary of what was done and what was found |
| Introduction | | |
| Background/rationale | 2 | (*✓*) Paragraphs 1, 2. |
| Objectives | 3 | (*✓*) Paragraph 3. |
| Methods | | |
| Study design | 4 | (*✓*)  (*✓*)  (*✓*) Subtitle “Study area and human sampling procedures” Paragraph 1.  (*✓*)  (*✓*) |
| Setting | 5 |  |
| Participants | 6 |  |
| Variables | 7 |  |
| Data sources/ measurement | 8 |  |
| Bias | 9 | (*✓*) Subtitle “Study area and human sampling procedures” Paragraph 1; Subtitle “Anthelmintic treatment and collection of expelled worms” Paragraph 1; and Subtitle “Serodiagnosis for cysticercosis” Paragraph 2. |
| Study size | 10 | (*✓*) Subtitle “Study area and human sampling procedures” Paragraph 1. |
| Quantitative variables | 11 | (*✓*) Fecal examination: Subtitle “Study area and human sampling procedures” Paragraph 1.  (*✓*) Serology ELISA: Subtitle “Serodiagnosis for cysticercosis” Paragraph 1. |
| Statistical methods | 12 | (*✓*) Subtitle “Data analysis”. |
| Results | | |
| Participants | 13 | (*✓*) Results Paragraph 1. |
|  |  |  |
| Descriptive data | 14 | (*✓*) Participants: Results Paragraph 2;  (*✓*) Parasitological examination: Subtitle “Fecal examination (KK), copro-PCR and tapeworm expulsion Paragraphs 1, 2.  (*✓*) Molecular diagnosis: Subtitle “DNA analyses from expelled tapeworms Paragraph 1.  (*✓*) Serology: Subtitle “Serological detection of cysticercosis Paragraphs 1, 2. |
| Outcome data | 15 | (*✓*) Treatment: Subtitle “Fecal examination (KK), copro-PCR and tapeworm expulsion Paragraph 2. |
| Main results | 16 | (*✓*) Parasitological examination: Subtitle “Fecal examination (KK), copro-PCR and tapeworm expulsion Paragraphs 1, 2.  (*✓*) Serology: Subtitle “Serological detection of cysticercosis Paragraphs 1, 2. |
| Other analyses | 17 | (*✓*) Subtitle “DNA analyses from expelled tapeworms” Paragraphs 1, 2. |
| Discussion | | |
| Key results | 18 | (*✓*) Summarise key results with reference to study objectives. |
| Limitations | 19 | (*✓*) Determination of worm carriers: Subtitle “MDA, diagnosis and the persistence of taeniasis and other neglected tropical diseases” Paragraph 2.  (*✓*) Serology: Subtitle “Cysticercosis education and hygiene” Paragraph 1.  (*✓*) Presence of “pure T. asiatica”: Subtitle “Intrinsic relationship between *T. saginata* and *T. asiatica*” Paragraph 1.  (*✓*) *T. asiatica* intermediate hosts: Subtitle “Intrinsic relationship between *T. saginata* and *T. asiatica*” Paragraph 1.  (*✓*) Necessity of more ecological studies: Subtitle “MDA, diagnosis and the persistence of taeniasis and other neglected tropical diseases” Paragraph 4. |
| Interpretation | 20 | (*✓*) Cultural issues: Subtitle “Ethnicity, general habits and parasitic infection in Sepon” Paragraph 1.  (*✓*) *T. asiatica* in Laos: Subtitle “Intrinsic relationship between *T. saginata* and *T. asiatica*” Paragraph 1, 2.  (*✓*) Other helminths found: Subtitle “MDA, diagnosis and the persistence of taeniasis and other neglected tropical diseases” Paragraph 4. |
| Generalisability | 21 | (*✓*) Discuss the generalisability (external validity) of the study results. |
| Other information | | |
| Funding | 22 | (*✓*) Described. |
